# Evaluation of the Structure of Myodural Bridges in an Equine Model of Ehlers-Danlos Syndromes

**DOI:** 10.1038/s41598-019-46444-w

**Published:** 2019-07-10

**Authors:** Abigail McElroy, Ann Rashmir, Jane Manfredi, Dodd Sledge, Elizabeth Carr, Edward Stopa, Petra Klinge

**Affiliations:** 1 Michigan State University College of Veterinary Medicine, Large Animal Clinical Sciences, East Lansing, MI USA; 2 Michigan State University College of Veterinary Medicine, Pathobiology and Diagnostic Investigation, East Lansing, MI USA; 3 Michigan State University Veterinary Diagnostic Laboratory, Lansing, MI USA; 4 Rhode Island Hospital, Departments of Pathology and Neurosurgery, Providence, RI USA; 5 Rhode Island Hospital, Department of Neurosurgery, Providence, RI USA

**Keywords:** Spinal cord, Translational research, Headache

## Abstract

Myodural bridges have been described in various species as connective tissue structures “bridging” small cranio-cervical muscles to the dura. Myodural bridges are thought to stabilize the dural sac during head and neck movements and promote cerebrospinal fluid motion; however, their role in neurological diseases has not yet been established. We report ultrasonographic visualization, necropsy, histopathologic and ultrastructural findings of myodural bridges in horses with hereditary equine regional dermal asthenia (HERDA), an equine model of Ehlers-Danlos syndromes. Five HERDA and 5 control horses were studied. Post-mortem examination and ultrasonographic studies (3 HERDA and 4 controls) demonstrated that the atlanto-occipital and atlanto-axial myodural bridges are dynamic structures “moving” the dura. En block resection of the myodural bridges (4 HERDA and 5 controls) was accomplished and histopathology showed myofiber degeneration in 3 HERDA horses and 1 control. Ultrastructural examination revealed loosely packed collagen fibrils with abnormal orientation in all HERDA horses compared to mild abnormalities in 2 controls. Our study provides necropsy and ultrasonographic evidence of the dynamic aspect of the myodural bridges as dural sac stabilizers. Myodural bridges may be pathologically altered in connective tissue disease as evidenced by the ultrastructural morphology in the HERDA myodural bridge.

## Introduction

Myodural bridges (MDBs) are bilateral dense bands of connective tissue that connect the suboccipital musculature with the dura mater at the atlanto-occipital (AO) and atlanto-axial (AA) levels^1–5^. They have been extensively studied in human cadavers and were first described by Hack *et al*. (1995)^1^. Scali *et al*. (2015) further pioneered detailed insight into these complicated structures using E12 sheet plastination in human cadavers with *in situ* visualization of those bridging structures^5^. The insight into the composition and arrangement of the posterior atlanto-occipital membrane and cerebrospinal dura was a break-through in the anatomy of the craniocervical junction and aided in the understanding of certain neurogenic disorders^5^. Myodural bridges have since been described in several animal species, including rhesus macaques, European rabbits, dogs, cats, Norway rats, guinea pigs, and the Indoasian finless porpoise^6^. According to both a recent thesis and a recent conference report, the existence of MDBs has been established in horses by cadaver studies^7,8^. The MDBs are theorized to stabilize the dura mater during extension of the head and neck, thus serving as dural tension monitors, preventing infolding of the dura mater and disruption of the flow of cerebrospinal fluid (CSF)^1,3–5,9–12^. The MDBs are also thought to function as a pump in conjunction with the suboccipital musculature to power CSF flow^13^. Myodural bridge dysfunction has been implicated as a cause of chronic headache and is theorized to be a contributor to the chronic headache phenotype observed in people with Ehlers-Danlos syndromes (EDS)^14–20^.

Our main objective was to further describe MDBs in horses, and to demonstrate that they are abnormal in horses with connective tissue disease. Horses with hereditary equine regional dermal asthenia (HERDA) were used as a naturally occurring animal model of EDS due to their similarities to people with kyphoscoliotic EDS (kEDS)^21,22^. Hereditary equine regional dermal asthenia is an autosomal recessive disorder that occurs in Quarter Horses and is caused by a mutation in peptidyl-prolyl cis-trans isomerase B (PPIB) encoding for cyclophilin B (Cyp B)^23^. Similar to people with EDS, horses with HERDA have fragile, hyperextensible skin as well as joint hypermobility^22,24^.

## Materials and Methods

### Animals

A total of 10 horses (5 HERDA and 5 control) were evaluated. The HERDA group consisted of 4 Quarter Horses and 1 Paint Horse. The HERDA group contained 2 juveniles (ages 2-years-old, 3-years-old), 2 adults (6-years-old, 10-years-old), and 1 geriatric horse (21-years-old), of which 3 were mares and 2 were geldings. The 10-year-old HERDA horse was not euthanized because it was client-owned; however, ultrasonographic examination was performed. The control horses consisted of 3 Quarter Horses, 1 Arabian, and 1 Morgan. The control group contained 3 juveniles (ages 2-years-old, 2-years-old, 3-years-old), 1 adult (6-years-old), and 1 geriatric horse (18-years-old), of which 4 were mares and 1 was a gelding. Control horses were donated due to non-neurologic health concerns which consisted of orthopedic and ophthalmic disease in this group. Genetic testing and physical examination were required for study enrollment for Quarter Horses and Paint Horses. Genetic testing was performed on 3 HERDA horses prior to study enrollment. All were confirmed to be homozygous positive, H/H, for the PPIB mutation via DNA testing conducted on hair root samples at UC Davis Veterinary Genetics Laboratory. The remaining 2 HERDA horses were tested using hair root samples at Michigan State University and were also homozygous positive, H/H, for the PPIB mutation. All HERDA horses displayed a phenotype consistent with the disorder, including skin hyperextensibility and fragility, abnormal scarring, and abnormal joint hypermobility. All 3 control Quarter Horses were confirmed to be homozygous negative, N/N, for the PPIB mutation via genetic testing on hair root samples at Michigan State University and were phenotypically normal. Carriers of the PPIB mutation, H/N, were excluded from the study. All horses used in this study were evaluated with the permission of the Michigan State University Institutional Animal Care and Use Committee. Experimental protocols were approved by, and performed in accordance with, the guidelines and regulations set forth by the Michigan State University Institutional Animal Care and Use Committee.

### Neurologic examinations

Static and dynamic neurologic examinations were performed on 3 HERDA horses and 4 control horses. Examination included observation of mentation, cranial nerve examination, assessment of spinal reflexes and muscle symmetry, and gait and postural assessment. Two HERDA horses were unable to be examined due to the early point at which they were evaluated. One control horse was unable to be examined due to a comminuted second phalangeal fracture.

### Ultrasonographic examination

A novel method for ultrasonographic examination of the MDBs was developed. Past studies in people have attempted to use MRI imaging to visualize the MDBs; however, ultrasound provides a more dynamic means of imaging. Prior MRI and ultrasonographic studies in horses have identified the AO membrane, but did not identify the MDBs^25,26^. Ultrasonographic imaging allows for visualization of motion of both the MDBs and the dura mater. Three HERDA horses and 4 control horses were imaged. Two HERDA horses and 1 control were not imaged due to the early point at which they were enrolled in the study, during which horses were solely assessed for presence of the MDB. For this study, 6 horses were imaged with a Mindray TE7 ultrasound and a 2–4 MHz cardiac probe. One horse was imaged with an Esaote ultrasound and a convex 1–8 MHz probe. To image the MDBs, frequency ranged from 3.4 to 4.2 MHz and depth ranged from 10 to 13 cm. An approximately 40 × 15 cm area was clipped and cleaned with alcohol. Horses were positioned with their heads in neutral position, which was defined as 105° ± 5°. Head position was measured with a HALO digital goniometer (HALO Medical Devices, Australia) at the angle between the ventral mandible and the jugular groove. Probe position and anatomic landmarks were selected based on past techniques developed for ultrasound-guided AO and AA centesis for CSF collection in standing horses;^25,27,28^ however, our imaging focused on the rectus capitis dorsalis minor (RCDmi) and the myodural attachments. Ultrasonographic imaging was conducted during head and neck movement between neutral, flexion, and extension to demonstrate the synchronized movements of the MDB with the dura mater. Flexion was defined as 90° ± 5°and extension was defined as 115 ± 5°.

In order to confirm that the structures viewed on ultrasound were in fact the MDBs, ultrasound guided injection of latex dye was performed at the AO and AA levels immediately following euthanasia in 2 control horses. An 18-gauge spinal needle was used to inject 2–3 mL of blue latex dye under ultrasound guidance.

### Anatomy of the equine myodural bridge

Four HERDA and 5 control horses were humanely euthanized to allow for anatomic examination of the MDBs. One HERDA horse, a 10-year-old Paint mare, was client-owned and was not euthanized for this study. Necropsy was performed within 1 hour of euthanasia in all but 1 control horse. Necropsy examination of one horse, a 6-year-old control, was performed at the Cummings School of Veterinary Medicine at Tufts University. All other necropsy examinations were performed at the Michigan State University Veterinary Diagnostic Laboratory. Bilateral AO and AA MDBs were successfully identified in all horses.

### Myodural bridge histology

The AO MDBs were collected at necropsy from 4 HERDA horses and 5 control horses and were submitted for histopathologic examination. Specimens were fixed for a minimum of 24 hours in 10% neutral buffered formalin and were routinely processed for histopathologic examination, embedded in paraffin, and sectioned at 5 *u*m. Resulting sections were stained with H&E, as well as Masson’s trichrome. Slides from 2 initial HERDA horses (aged 2-years and 3-years-old) and 1 control horse (6-years-old) were processed at Rhode Island Hospital Department of Neuropathology and photographed with a SPOT Insight 2Mp Monochrome FireWire Digital Camera and SPOT imaging software. All other specimens were processed at the Michigan State University Veterinary Diagnostic Laboratory where they were photographed with an Olympus BX45 microscope with an Olympus DP71 camera and Olympus cellSens software. Efforts were made to section all specimens in the same orientation, and to include the RCDmi, MDB, and dura mater in each section.

CD3 (T cells) and CD20 (B cells) immunohistochemistry (IHC) was performed on 4 HERDA and 5 control MDBs to better evaluate the types of cells present. Tissues were formalin fixed and paraffin embedded. Dewaxing was performed with Bond Max DeWax (Leica; Wetzlar, Germany). Sections were stained using a Bond-Max autostainer (Leica; Wetzlar, Germany). Slides were incubated with rabbit anti-human polyclonal anti-CD20 antibody (RB = 9013-P; Thermo Scientific, Rockford, Illinois) for B cells at a dilution of 1:200 for 30 minutes and with rabbit anti-human polyclonal anti-CD3 antibody (A0452; DAKO, Carpinteria, California) for T cells at a dilution of 1:200 for 30 minutes^29–31^. These antibodies are validated for use in horses, and equine lymph node was used as a positive control^32^. Antigen retrieval was accomplished via immersion in a citrate buffer for 30 minutes, while 3,3′-Diaminobenzidine was used as a chromogen and hematoxylin as a counterstain. Bond Polymer Refine Detection (Leica; Wetzlar, Germany) was the IHC kit used for both CD3 and CD20. All IHC was performed at the Michigan State University Veterinary Diagnostic Laboratory.

### Myodural bridge ultrastructure

The AO MDBs from 4 HERDA and 5 control horses were submitted for transmission electron microscopy. Connective tissue from the cranioventral aspect of the bridge, adjacent to the junction of the dense connective tissue and dura mater, was submitted for processing. Samples were formalin fixed for 24 hours prior to transfer to glutaraldehyde in preparation for TEM processing at Michigan State University Center for Advanced Microscopy. Two samples, 1 HERDA horse and 1 control, were removed from paraffin blocks, due to a lack of tissue left in formalin after histopathology. Due to the durability of connective tissue, this method was deemed acceptable. After primary fixation in glutaraldehyde, samples were washed with 0.1 M cacodylate buffer. Samples were then postfixed with 1% osmium tetroxide in 0.1 M cacodylate buffer, dehydrated in a gradient series of acetone, and infiltrated and embedded in Spurr^33^. A Power Tome Ultramicrotome (RMC, Boeckeler Instruments; Tucson, AZ) was used to cut 70 nm thin sections and post stained with uranyl acetate and lead citrate. One HERDA horse sample was processed at Rhode Island Hospital where upgraded alcohols were used for dehydration followed by four changes of propylene oxide. This sample was then embedded in Epon-Araldite rather than Spurr, prior to sectioning, staining, and imaging at Michigan State University Center for Advanced Microscopy^34^. No other deviations from protocol were introduced for this sample. All other samples were imaged at Michigan State University Center for Advanced Microscopy. The MDB was imaged at 14,000×, 27,000×, and 40,000× in longitudinal and cross-section using a JEOL 100CX Transmission Electron Microscope (Japan Electron Optics Laboratory, Japan) at an accelerating voltage of 100 kV. Imaging at higher or lower magnification was performed when indicated.

### Sarcocystis neurona testing

Cerebrospinal fluid was obtained via centesis of the cerebellomedullary cistern immediately following euthanasia in 2 HERDA horses and 4 controls and was stored at −80 °C for future molecular analysis. Cerebrospinal fluid from 1 HERDA horse, a 21-year-old mare, was submitted for a western blot for antibody to *Sarcocystis neurona* at the Michigan State University Veterinary Diagnostic Laboratory due to the presence of sarcocysts in the RCDmi and abnormalities in the neurologic examination.

## Results

### Neurologic examinations

The results of all control horse examinations were within normal limits. Abnormalities were noted in 1 HERDA horse, a 21-year-old Quarter Horse mare, that displayed mild truncal sway and a base-narrow, single-track gait in the hind limbs when blindfolded with the head elevated. This horse had cervical spine osteoarthritis, and this gait may be attributed to cervical spine pathology; however, an additional lumbosacral lesion cannot be ruled out. Equine protozoal myeloencephalitis due to *S*. *neurona* was excluded due to a negative western blot performed on CSF; however, disease due to *Neospora hughesi* is still possible.

### Ultrasonographic examination

**Figure 1:**
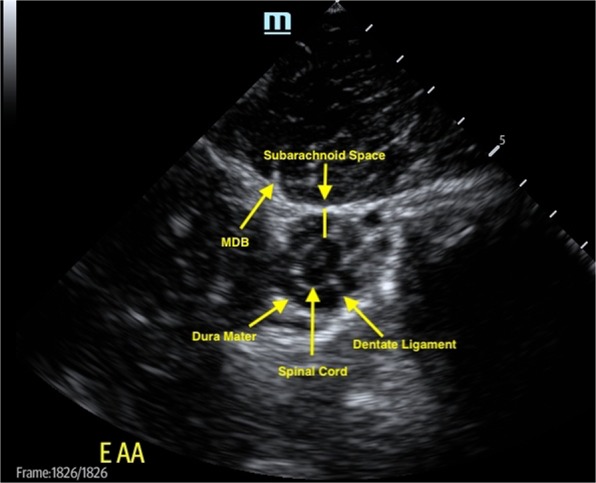
Ultrasonographic anatomy of the equine MDBs at the AA level from a 2-year-old control Arabian mare. The spinal cord, dura mater, dentate ligaments, MDBs, and subarachnoid space are visible.

**Figure 2:**
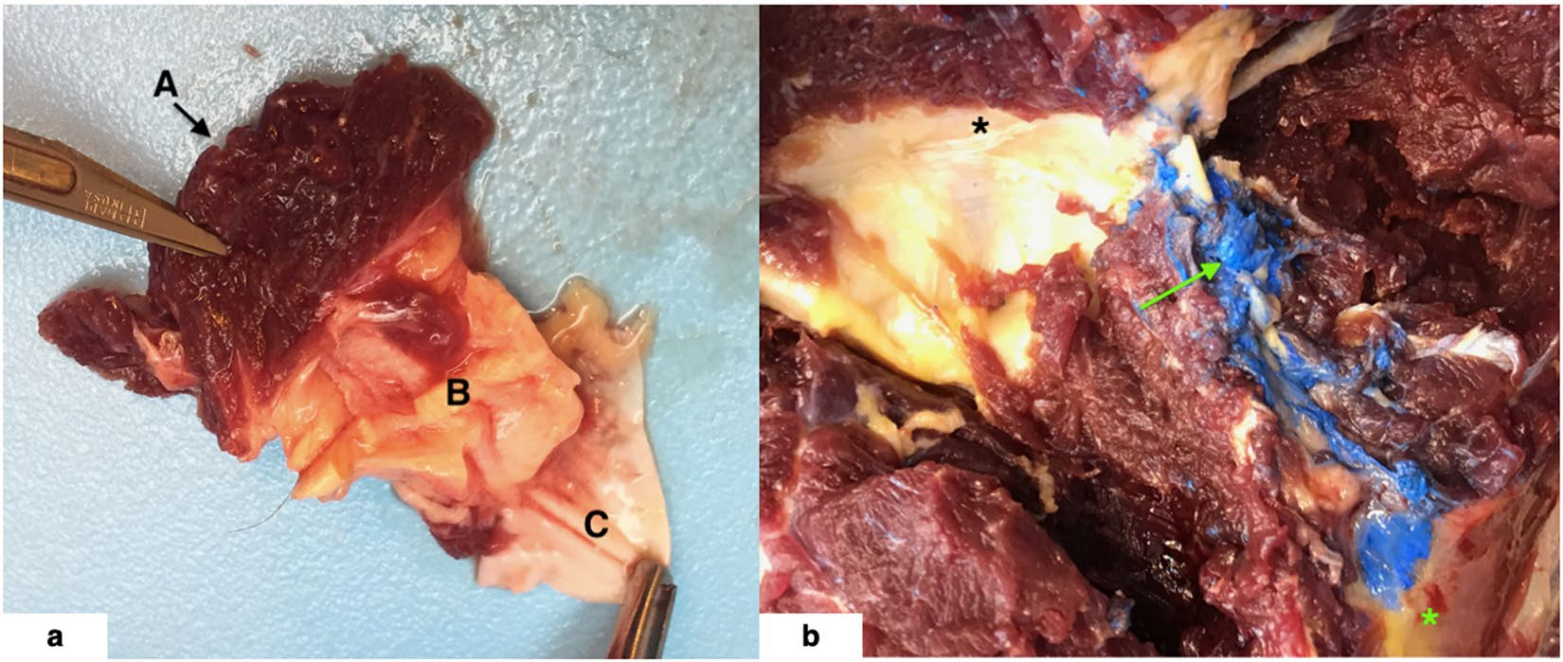
(**a**,**b**) 2a = En bloc dissection of the equine AO MDBs from aged HERDA horse. A = RCDmi, B = dense connective tissue complex of the MDBs including the dorsal atlanto-occipital membrane, and C = dura mater. 2b = Atlanto-axial MDB () labelled with blue latex dye via ultrasound-guided injection from one aged control horse. The AA MDBs spans from C1 (*) to C2 ().

The MDBs, dura mater, dentate ligaments, subarachnoid space, and spinal cord were easily visible at both the AO and AA interspaces (Fig. 1). Dural movement was observed as a result of head and neck movement during ultrasonographic examination (Video file 1). At necropsy, the presence of latex dye within the AO and AA MDBs following ultrasound-guided injection confirmed the identity of the structures identified as the MDBs on ultrasound in 2 control horses (Fig. 2b).

### Post mortem findings of the equine myodural bridges

Myodural bridges were discovered in the horse at both the AO and AA levels. At the AO level, the MDBs originated from the RCDmi. En block resection of the AO and AA MDBs was accomplished in all horses (Fig. 2a) and manipulation of the MDBs via traction on the RCDmi and rectus capitis dorsalis major (RCDma) was observed to tent the dura. The MDBs attached via the dorsal atlanto-occipital membrane, a dense connective tissue complex, to the spinal dura. At the AA level, the MDBs originated from the RCDma and obliquus capitis caudalis. The equine nuchal ligament was not found to contribute to the MDBs. No gross anatomical difference in MDB anatomy was observed between horses. Osteoarthritis was noted during necropsy at the AO and AA levels in only 1 HERDA horse, a 21-year-old mare.

### Myodural bridge histology

**Figure 3:**
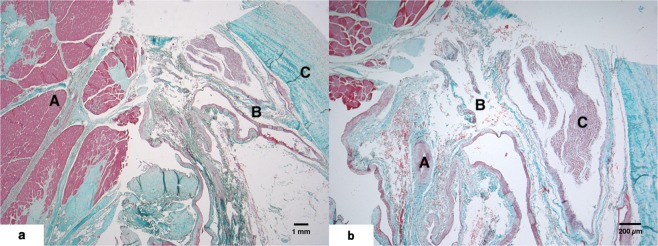
(**a**,**b**) 3a = Six-year-old control Morgan horse AO MDB stained with Masson’s trichrome. A = RCDmi, B = connective tissue, C = dura mater. 3b = On higher magnification one could identify vasculature (A), adipose tissue (B) a peripheral nerve associated with connective tissue core of AO MDB (C).

The MDBs were composed of areas of dense connective tissue interspersed with loose connective tissue, nervous tissue, adipose tissue, and vasculature (Fig. 3a,b). Synovia were present along the outer edges of the MDB in equine specimens as a result of dissecting along the lateral masses and facets of the AO and AA junctions.

**Figure 4:**
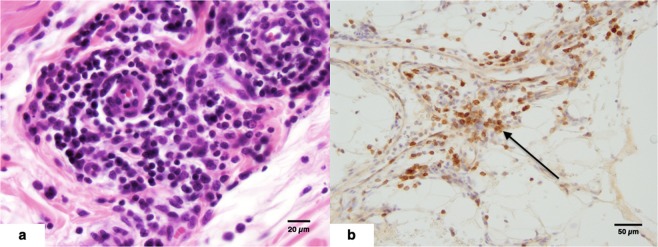
(**a**,**b**) 4a = Lymphocytic and plasmacytic perivascular cuffing in a 3-year-old HERDA horse gelding MDB stained with H&E. 4b = CD3 IHC demonstrating loose perivascular to scattered interstitial lymphocytic infiltrates (→) in a 3-year-old HERDA horse gelding MDB.

Inflammatory changes were present in 1 HERDA horse MDB. This horse, a 3-year-old Quarter Horse, displayed large aggregates of lymphocytes, as well as lymphocytic and plasmacytic perivascular cuffing. The presence of both T and B lymphocytes was confirmed via CD3 and CD20 IHC (Fig. 4a,b). The remaining 8 horses, 3 HERDA horses and 5 controls, displayed none or a few scattered T lymphocytes in the MDB vessel walls, changes which were believed to be within normal limits.

Myofiber degeneration was observed in the RCDmi in 3 HERDA horses and 1 control. A 21-year-old HERDA mare had myofiber degeneration with secondary phagocytosis of necrotic material by macrophages, as well as multiple sarcocysts within the muscle. Due to the presence of sarcocysts, CSF was submitted for a western blot to test for *S*. *neurona* antibodies; results were negative. The sarcocysts were suspected to be *Sarcocystis fayeri*, a commonly occurring sarcocyst incidentally found in the muscle of horses. No evidence of sarcocyst rupture was observed. Lack of periodic acid-Schiff staining ruled out polysaccharide storage myopathy as a cause of myofiber degeneration. A 6-year-old HERDA mare displayed rare degenerate myofibers with phagocytosis of necrotic material by macrophages and mild infiltrates of eosinophils in the RCDmi. A single sarcocyst was observed within the RCDmi of this horse. A third HERDA horse, a 3-year-old gelding, had a focal area of myofiber degeneration with macrophage infiltration. The musculature was normal in all but one control, a 3-year-old Quarter Horse gelding that had extensive necrosis of myofibers and replacement by macrophages within the RCDmi. Additionally, it was observed at necropsy that this horse had a grossly visible area of necrosis in the superficial cervical musculature. On histology, the semispinalis capitis was found to have a focal area of myofiber necrosis and extensive phagocytosis of necrotic debris by macrophages. This horse had a history of trauma sustained during trailering.

### Myodural bridge ultrastructure

**Figure 5:**
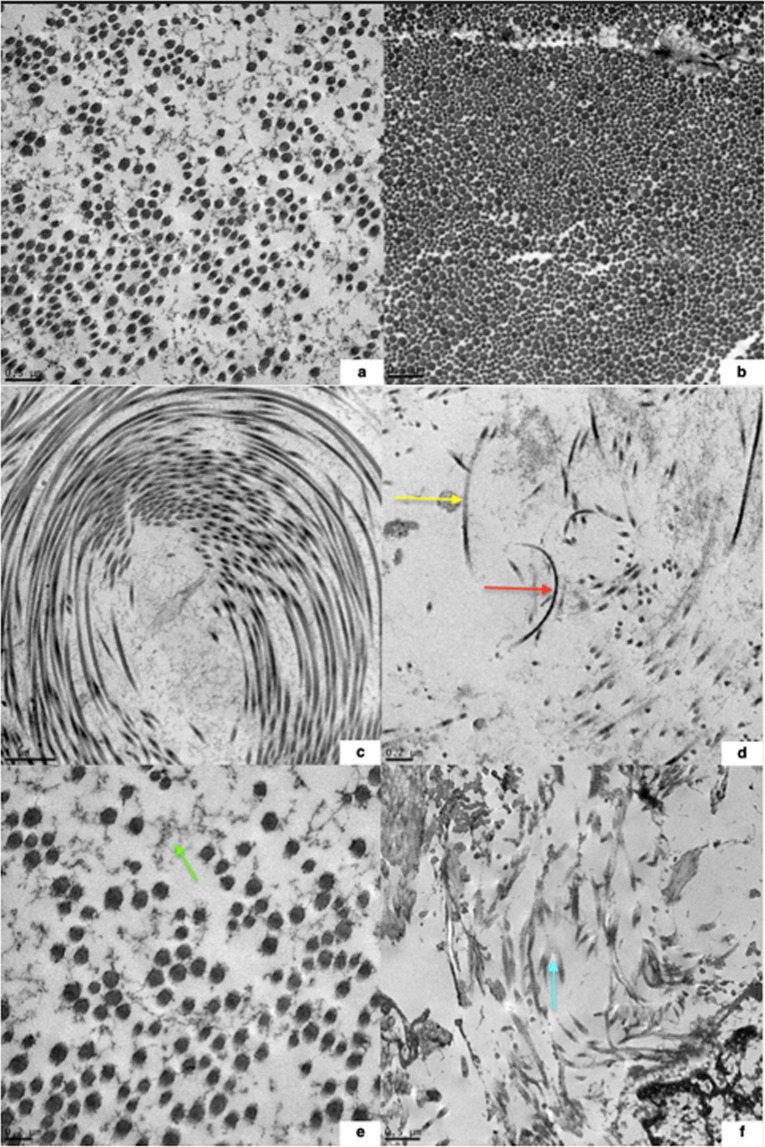
(**a**–**f**) Comparison of density of collagen fibril packing in cross-section in age-matched HERDA vs. control horse MDBs and abnormal collagen fibril orientation in the MDBs of HERDA horses imaged with TEM. 5a = Three-year-old HERDA horse gelding with loosely packed collagen fibrils, mild variation in fibril size, variation in fibril shape, and granulo-filamentous deposits in the interfibrillar space. 5b = Two-year-old control Quarter Horse mare with densely packed collagen fibrils and variation in fibril size. 5c = Whirling collagen fibrils in a 3-year-old gelding. 5d = Hook shaped () and fragments of () collagen fibrils, as well as loosely packed collagen fibrils in a 6-year-old mare. 5e = Granulo-filamentous deposits () in the interfibrillar space, as well as loosely packed collagen fibrils in a 3-year-old gelding. 5 f = Fragmented () and loosely packed collagen fibrils in a 2-year-old gelding.

Differences in collagen fibril size, fibril packing density, and fibril orientation were subjectively compared between HERDA and control horses. The results are summarized in Table 1. In cross-section, 3 out of 4 HERDA horses had large areas of loosely packed collagen fibrils. None of the control horses displayed such areas; collagen in the control horses was predominately densely packed with rare areas of loosely packed fibrils (Fig. 5a,b). Also, moderate to severe abnormalities in collagen fibril orientation were only observed in the HERDA horses, with 3 out of 4 HERDA horses displaying prominent abnormalities in fibril orientation, such as whirling collagen and hook shaped or fragmented features (Fig. 5c–f). Only 2 of 5 control horses showed abnormalities in fibril orientation, which were mild with only rare disrupted fibrils and were felt to be normal. Variation in fibril size and shape was found among HERDA and control horses of all ages, suggesting that this might be a normal finding in the equine MDBs. Granulo-filamentous deposits were observed in two HERDA horses and not in the controls.

**Table 1:** Findings from TEM imaging of the MDB of HERDA and control horses.

	Collagen Fibril Size and Shape in Cross-Section, Fibril Packing Density	Abnormal Collagen Fibril Orientation	Granulo-filamentous Deposits
HERDA 1	Variation in collagen fibril size and shape, densely packed collagen fibrils	No	No
HERDA 2	Variation in collagen fibril size and shape. Loosely packed collagen fibrils, with no areas of dense packing present	Hook-shaped collagen fibrils, fragmented collagen fibrils	Yes
HERDA 3	Normal collagen fibril size with mild variation in shape. Focal areas of loosely packed collagen fibrils present	Whirling collagen fibrils	Yes
HERDA 4	Normal collagen fibril size with mild variation in shape. Focal areas of loosely packed collagen fibrils present	Hook-shaped collagen fibrils, fragmented collagen fibrils	No
Control 1	Normal collagen fibril size and shape. Focal areas of loosely packed collagen fibrils	Rare fragmented collagen fibrils	No
Control 2	Variation in collagen fibril size and shape, densely packed collagen fibrils	No	No
Control 3	Variation in collagen fibril size and shape. Focal areas of loosely packed collagen fibrils	Rare hook-shaped and fragmented collagen fibrils	No
Control 4	Variation in collagen fibril size and shape, densely packed collagen fibrils	No	No
Control 5	Variation in collagen fibril size and shape, densely packed collagen fibrils	No	No

## Discussion

The results of this study support that MDBs are a conserved anatomic structure across mammalian species^6^. Our study provides necropsy (post-mortem) and ultrasonographic findings that are directed to the dynamic aspect of the MDBs as dural sac stabilizers in horses. Further, this is the first study to report that MDBs may be pathologically altered in disease and can specifically be pathologically altered in connective tissue disease as evidenced by the ultrastructural histology in the HERDA MDB. Severe ultrastructural abnormalities in the collagen fibril orientation were present only within the HERDA MDB but not in the controls. It is conceivable that the function of the MDBs is impaired in HERDA horses, and we speculate in EDS patients as well.

While horses with HERDA are known to have many similarities to people with EDS, the neurologic status of HERDA horses has not previously been documented. The anatomy of the equine craniocervical region shares many similarities with people, making them an excellent model for examination of the MDBs^35^. At the AO level, the equine MDBs originate from the RCDmi, a muscle analogous to the RCPmi in people^35^. As in people, the MDBs attach via the posterior, or dorsal, atlanto-occipital membrane-spinal dura complex^1,5,36^. At the AA level, the MDBs originated from the RCDma, a muscle analogous to the RCPma in people. The obliquus capitis caudalis, analogous to the obliquus capitis inferior in people, also contributes to the MDB in the horse^35^. The anatomy of the equine MDB attachments at the AO and AA levels is virtually identical to that of other land-dwelling mammals, including the attachment through the dorsal atlanto-occipital membrane-spinal dura complex at the AO level. This anatomy differs from the description by Zheng, *et al*. (2017) of the Indoasian finless porpoise, a marine mammal with an AO MDB which does not attach via the posterior atlanto-occipital membrane-spinal dura complex, and instead inserts directly onto the dura mater via tendon like projections from the RCDmi. The Indoasian finless porpoise also has a muscular attachment at the AA level which includes the lateral rectus capitis dorsal muscle, a muscle not found in the land-dwelling mammals described by Zheng *et al*.^6^.

Ultrasonography gives dynamic insights into how the anchoring elements of the spine function, the intradural filaments and emerging spinal nerve roots stabilize and center the spinal cord inside the dural sac. The extradural MDBs connect the dura to the surrounding head and neck muscles. This study corroborates the likelihood that MDBs allow the dural sac to remain patent even with neck and head movement. This physiological concept has been previously suggested based on histologic and necropsy findings in people^9^ and is supported by our own *in*-*vivo* ultrasound imaging showing all anatomical elements involved in the human study, the nerve roots, the dentate ligaments, the dural sac, and the MDBs. Ultrasonographic imaging in horses should encourage respective studies of the cervical spine in people to evaluate the configuration of the dural sac during head and spine movement.

Histologically, the human and equine MDB share many similarities. The MDBs of both species contain connective tissue, adipose tissue, vasculature, and peripheral nervous tissue^10,11^. The ultrastructure of the MDB has not yet been examined in people; however, there is a large body of work examining the ultrastructure of other tissues, particularly skin, in the EDS population. People with EDS often display collagen fibrils described as whirling within bundles, as well as hook-like or twisted fibrils. Collagen fibrils with irregular edges in cross-section, as well as loosely packed collagen fibrils, are also common findings in EDS^37^. Similar findings have been described in the skin and corneas of HERDA horses^24,38^. The HERDA MDBs examined in this study displayed characteristics, such as decreased fibril packing density and abnormal fibril orientation, that are commonly observed in the skin of people with EDS. Additionally, granulo-filamentous deposits were observed in the interfibrillar space in the HERDA population, which are commonly associated with kEDS in people^21,37^. The abnormal ultrastructural collagen fibril orientation seen in HERDA horses but not controls may suggest “defective” MDB dynamics in HERDA. A recent article by Zheng *et al*. (2018) of anatomical and histologic analyses of AO and AA MDBs in human cadavers did show that MDBs in fact display parallel collagen fibers. This group used a method sensitive to fibril orientation, the “fiber quad staining” method, to detect reticular fibers, elastic fibers and collagen fibers. They found that the MDB was mainly made of collagen type 1 fibers, fibers which are most common, and observed in tendons as well. These authors suggest that MDBs are high-tensile tendon-like structures^39^.

Peripheral nerves in the MDBs have also been described in human cadaver histology, and this study was able to demonstrate the presence of peripheral nerve associated with the connective tissue core of the equine AO MDB via histopathology^10,11^. This finding further supports an anatomical and functional significance, as well as the clinical relevance of the myodural bridges’ function as a passive and active stabilizer of the cervical spinal cord across species. The active contribution of the MDBs in the neuromuscular stabilization of the spinal cord has been previously discussed by Scali, Pontell and Enix *et al*. (2011, 2013, 2014, 2015) as evidenced by the presence of neural tissues within the MDB structures using IHC staining with anti-neurofilament protein antibodies^3–5,10–12^.

Further ultrasonographic studies are being conducted to evaluate the presence of dural infolding secondary to MDB dysfunction in the equine HERDA and human EDS populations, which could be conducted by measuring the subarachnoid space with the head and neck positioned in neutral, flexion, and extension. Additionally, CSF was banked for future analysis to allow comparison of equine HERDA and human EDS CSF for markers of inflammation and collagen breakdown products, such as pyridinoline and deoxypyridinoline^21^.

A limitation of this study was our sample size. Although our sample size of 5 horses and 5 controls is admittedly small, it is in keeping with many of the recent referred publications on HERDA^40–42^. Additionally, horses with HERDA can be a difficult population of horses to obtain and maintain.

Although our controls and HERDA cases are not perfectly sex matched we do have representative horses in each group. In the control horses there were four mares and one gelding while in the HERDA horse population there were three mares and two geldings. Further support for a lack of impact due to this discrepancy comes from the literature where Zumpano *et al*. (2006) found that sex differences in people do not impact the MDB^36^.

One true limitation of our study is our inability to accurately assess horses, and for that matter most species, for headaches. Unfortunately, there are no tests for head pain in the horse. Also, the significance of the mildly abnormal neurological exam in one HERDA horse is unclear. Osteoarthritis in this animal was observed throughout the cervical spine during ultrasonographic examination, which is a clinically appropriate imaging modality to address this issue^43^. Radiographs of the cervical spine were not obtained because we were able to directly assess the spine at necropsy. A single negative western blot on CSF in this horse is within the standard of practice to rule out EPM in veterinary medicine. The sensitivity and specificity for the western blot on CSF both approach 89%^44,45^.

All horses had the same procedures performed (neurologic examination, ultrasound, histopathology, transmission electron microscopy) with few exceptions. The initial horse did not receive a neurologic or ultrasonographic examination but was not noticeably neurologic upon enrollment. This horse presented with a phalangeal fracture and could not be subjected to additional testing prior to euthanasia. We felt it was still appropriate to use this horse to establish necropsy and histopathologic techniques before commencing the larger study. One horse did not undergo histopathology or transmission electron microscopy as it was client owned and not euthanized. The necropsy of one control horse occurred more than 1 hour after being euthanized but did not display histological disintegration so it was included in the study. The high fat content in some of the tissues and lack of enzymatic activity in all tissues studied make them more resistant to degradation when compared to tissues such as the pancreas or stomach. Also, ultrasonographic studies were only added once we were confident about the structure of the equine MDBs after several horses were euthanized.

We feel that the strength of our study lies in its thorough descriptive nature. Evidence for the presence of abnormalities in the HERDA horse MDB by virtue of anatomic and dynamic validation via necropsy, ultrasound and ultrastructural techniques indicate that MDBs are abnormal in HERDA horses when compared to controls.

### Conclusions and clinical research implications

The ultrastructural findings observed in the HERDA MDB, such as decreased collagen fibril packing density and abnormal collagen fibril orientation, suggest decreased integrity of connective tissue. This study is a step towards better understanding the role of MDBs in human disease(s). Further ultrasonographic studies in horses and people with connective tissue disease might determine the impact of MDB action on subarachnoid space width and correlation with clinical symptoms, i.e. headaches and craniocervical neurological symptoms.

## Supplementary information

Equine Atlantoaxial Ultrasonographic Examination

## Data Availability

The datasets generated during the current study are available from the corresponding author upon reasonable request.
